# Haloperidol’s Cytogenetic Effect on T Lymphocytes of Systemic Lupus Erythematosus and Rheumatoid Arthritis Patients: An In Vitro Study

**DOI:** 10.7759/cureus.42283

**Published:** 2023-07-21

**Authors:** Georgios Demirtzoglou, Sofia-Ifigeneia Chrysoglou, Zafeiroula Iakovidou - Kritsi, Alexandros Lambropoulos, Alexandros Garyfallos

**Affiliations:** 1 1st Laboratory of Medical Biology and Genetics, School of Medicine, Faculty of Health Sciences (FHS) of Aristotle University of Thessaloniki, Thessaloniki, GRC; 2 Department of Rheumatology, 251 General Airforce Hospital, Athens, GRC; 3 4th Department of Internal Medicine, Hippokration General Hospital, Thessaloniki, GRC

**Keywords:** systemic lupus erythematosus, mitotic index, proliferation rate index, t lymphocytes, rheumatoid arthritis, cytogenetic studies, sister chromatid exchanges, haloperidol

## Abstract

Objectives: Investigating haloperidol’s cytogenetic behavior in cultured human T lymphocytes of patients with systemic lupus erythematosus (SLE) and rheumatoid arthritis (RA).

Methods: Four haloperidol solutions were added in cultures of peripheral blood lymphocytes of healthy individuals, SLE, and RA patients. After 72 hours of incubation, the cultured lymphocytes were plated on glass slides, and stained with the fluorescence plus Giemsa method, and sister chromatid exchanges (SCEs), proliferation rate index (PRI), and mitotic index (MI) were measured with the optical microscope.

Results: Result analysis revealed: (a) a statistically significant (p=0.001) dose-dependent increase of SCEs in SLE patients compared to healthy individuals; (b) a statistically significant (p=0.001) dose-dependent decrease of SCEs in RA patients for haloperidol concentrations 5, 10μg/mL; (c) a statistically significant (p=0.001) dose-dependent increase of SCEs in RA patients for haloperidol concentrations 20, 100μg/mL; and (d) a statistically significant (p=0.001) dose-dependent reduction of PRI and MI in both patient groups compared to healthy individuals. Furthermore, a correlation was observed between (a) SCE and PRI index variations, (b) MI and SCE index variations, and (c) PRI and MI index variations.

Conclusions: Haloperidol affects T lymphocytes from SLE and RA patients by modifying DNA replication procedures, DNA damage response, and ferroptosis. Considering the wide use of haloperidol in neuropsychiatric symptoms of SLE and RA patients, further studies with more immune cell subsets are needed to evaluate its effects on human genetic material.

## Introduction

Haloperidol, a typical antipsychotic drug, is indicated for treating acute psychosis, including schizophrenia, bipolar disorder, and other psychiatric disorders. It is also approved for Tourette syndrome treatment. It is off-label and used for hiccup treatment. Haloperidol exerts antipsychotic activity by antagonizing the D2 dopamine receptors [1].

Systemic lupus erythematosus (SLE) is a chronic, multisystem autoimmune disorder that typi­cally affects women of child-birthing age. It can affect almost any organ, including the central nervous system and the kidneys. Diagnosis can be difficult because of SLE heterogeneity, and it is primarily clinical. T lymphocytes and DNA damage are critically involved in SLE pathogenesis [2].

Rheumatoid arthritis (RA) is a chronic, inflammatory, autoimmune disease that primarily affects the joints causing synovial tissue damage and disability [3]. T lymphocyte abnormalities are detected in the early stage of RA development. DNA damage, overall genomic instability, and metabolic defects of T lymphocytes are associated with RA pathogenesis [4].

Haloperidol’s cytogenetic behavior has been studied in vitro in lymphocytes from healthy individuals [1,5] and in vivo in lymphocytes from schizophrenia patients [6,7]. Conflicting results arise from both in vitro and in vivo haloperidol studies. Zamani et al. demonstrated a cytotoxic effect of haloperidol in vitro in lymphocytes from healthy donors [1]. In contrast, Gajski et al. found no cytotoxic cytogenetic behavior of haloperidol, but they indicate that it may exert T lymphocyte cytostaticity [5]. Zampas, in 2015, concluded that haloperidol appears to be both cytotoxic and cytostatic in vivo in schizophrenic patients [6]. Still, Al Eitan et al. found no evidence of cytotoxic behavior of haloperidol in vivo in lymphocytes from schizophrenic patients [7]. Haloperidol’s cytogenetic behavior in lymphocytes from patients with autoimmune diseases has not yet been studied. It is important because haloperidol may exert immunomodulatory-antirheumatic actions by influencing cytokine secretion [8,9]. Grimaldi showed that long-term low-dose haloperidol may act like hydroxychloroquine in modifying RA clinical and laboratory parameters [10].

The aim of the present study is to evaluate the effect of haloperidol in T lymphocyte DNA of patients with SLE and RA by estimating three sensitive cytogenetic indices: Sister chromatid exchanges (SCEs), proliferation rate index (PRI), and mitotic index (MI). SCEs are defined as the exchange of genetic material between two identical sister chromatids, and they occur during the DNA replication procedure due to unrepaired mistakes. The SCE index has been identified as one of the most sensitive indices among sensitive biomarkers of genotoxicity, together with chromosomal aberrations, comet assay, and micronuclei [11]. PRI and MI have been used as sensitive indicators for evaluating the cytostatic activity of various environmental hazards or therapeutic agents [11,12]. Changes in SCE frequency, PRI, and MI, represent the first signs of DNA damage [11,12].

## Materials and methods

Ethics statement

The ethics committee of Aristotle University of Thessaloniki School of Medicine approved the study (approval number: 9.657/12-7-2017). Written informed consent was obtained from all individuals.

Patients and controls

The present study enrolled 30 SLE patients (25 females, 5 males) and 30 RA patients (20 females, 10 males) from the Rheumatology Clinic of the 4th Department of Internal Medicine of Hippokaration Hospital. SLE patients met either the revised 1997 ACR classification criteria for SLE or the 2012 Systemic Lupus International Collaborating Clinics (SLICC) classification criteria for SLE. RA patients were meeting the 2010 American College of Rheumatology (ACR)/European League Against Rheumatism (EULAR) RA Classification Criteria. Patients did not receive haloperidol as part of their treatment. SLE patients’ mean age was 28±7 years, they were all anti-nuclear antibodies (ANA) positive, and 60% were anti-double strand DNA (anti-dsDNA) positive at the time of diagnosis. RA patients’ mean age was 47±6 years; they were all either RF-positive or ACPA-positive. All patients had achieved disease remission or low to moderate disease activity. Disease activity was measured with Systemic Lupus Erythematosus Disease Activity Index (SLEDAI) score for SLE patients and DAS28 for RA patients. All patients were treated with conventional synthetic Disease-Modifying Anti-Rheumatic Drugs (DMARDs) with or without a low dose of prednisone, and 60% of RA patients were treated with a TNF-a inhibitor.

The control group comprises 30 healthy non-smokers (15 females, 15 males, mean age 20±2 years). They did not show any signs of infection in the past 15 days and did not consume any medication. Venous blood (5-7mL) collected from all the above-mentioned individuals was used immediately for lymphocyte culture preparation.

SCE, PRI, and MI assays

Human lymphocyte cultures [11,12] were prepared by adding in 5 mL chromosome medium (RPMI-1640, Biochrome, supplemented with 20% fetal calf serum, 0.63% L-glutamine, 0.63% penicillin/streptomycin, and 2% phytohaemagglutinin) at the beginning of the culture life 11-12 drops of heparinized whole peripheral blood, 5-Bromodeoxyuridine (BrdU) solution (5μg/mL/culture) and haloperidol solution (A=5μg/mL or B=10μg/mL or C=20μg/mL or D= 100μg/mL).

T lymphocyte cultures were incubated at 37°C for 72 hours in a dark incubator to minimize photolysis of BrdU. Colchicine was added 2h before the end of the incubation. T lymphocytes were then collected by centrifugation and exposed to 0.075M KCl (potassium chloride) solution for 12 minutes. The hypotonic solution spreads chromosomes and causes hemolysis of red blood cells. The pellet was fixed three times with methanol: acetic acid (3:1) solution. Drops of a concentrated suspension of cells were placed on micro slides and allowed to air dry. For SCE, PRI, and MI analysis, the slides were stained by a modification of the fluorescence plus Giemsa procedure to obtain harlequin chromosomes [11,12].

Statistical analysis

For SCEs estimation, 30 suitably spread second-division nuclei from each culture were blindly scored. For PRI calculation, 100 nuclei in each culture's first, second, third, or higher divisions were blindly scored. PRI=M1+2M2+3M3/100, where M1, M2, and M3+ are the percent values of nuclei in the first, second, third, or higher divisions, respectively. All cell divisions present in an optical field of 1000 nuclei were scored for MI analysis. MI = number of cells in mitosis/total number of nuclei (1000). The statistical analysis was done using the Statistical Product and Service Solutions (SPSS) (IBM SPSS Statistics for Windows, Version 22.0, Armonk, NY) statistical package. All values were expressed as mean ± standard error of the mean (SEM). Comparison of values between the different groups and subgroups was accomplished by the nonparametric Mann-Whitney U-test. The Kruskal-Wallis test was used to evaluate the dosage effect of haloperidol on cytogenetic indices. Spearman's rank correlation coefficient was applied to calculate the correlation between SCEs, PRI, and MI.

## Results

**Figure 1:**
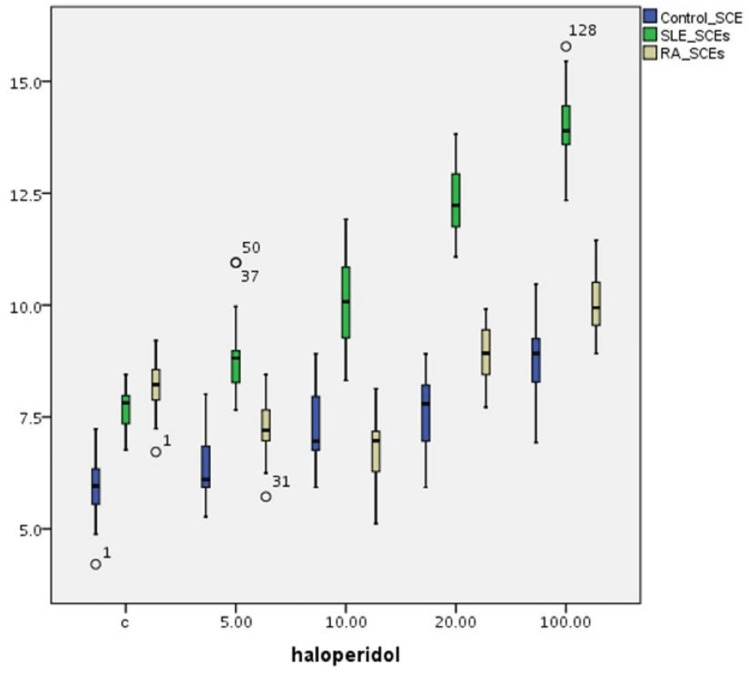
Haloperidol’s (μg/mL) effect on SCE index (SCE/cell±SE) variations in human T-lymphocyte cultures from healthy individuals, SLE, and RA patients SCE: sister chromatid exchange; SLE: systemic lupus erythematosus; RA: rheumatoid arthritis

Table 1 summarizes the effect of the four haloperidol concentrations on SCE index variations on T-cultured lymphocytes of all three groups (Figure 1). There is a statistically significant difference in SCE frequencies between (a) the control and the SLE group (p=0.001), (b) the control and the RA group (p=0.001), and (c) the SLE and RA group (p=0.001) without the effect of haloperidol. The cultured lymphocyte SCE frequencies of the SLE patient group are statistically significantly increased (p=0.001) compared to those of the control group for all haloperidol concentrations. Cultured lymphocytes SCE frequencies of the RA patient group are statistically significantly increased for all haloperidol concentrations (p<0.001) compared to the control group except for 10μg/mL haloperidol concentration. For cultured lymphocytes of the control group, a statistically significant induction in SCE frequency is observed for all haloperidol concentrations (p=0.001). Furthermore, a dose-dependent induction in SCE frequency is observed in the SLE group (p=0.001). In contrast, a reduction in SCE frequency for 5 and 10μg/mL haloperidol concentrations in the RA group is observed (p=0.001). An increase in SCE frequencies is noticed at higher (20 and 100μg/mL) haloperidol concentrations (p=0.001).

**Table 1:** Haloperidol’s effect on SCE frequency (SCE/cell±SE) in T lymphocyte cultures of SLE, RA patients, and healthy individuals p^1^ represents the p-value for the Kruskal-Wallis test, which was performed to evaluate the dosage effect of haloperidol on SCE for all groups, p^2^ represents the p-value for the Mann-Whitney U test for the comparison of the SCE index between control and SLE group for various haloperidol concentrations, p^3^ represents the p-value for the Mann-Whitney U test for the comparison of the SCE index between control and RA group for various haloperidol concentrations, p^4^ represents the p-value for the Mann-Whitney U test for the comparison of the SCE index between RA and SLE group for various olanzapine concentrations. SCE: sister chromatid exchange; SE: standard error; SLE: systemic lupus erythematosus; RA: rheumatoid arthritis

Haloperidol (μg/mL)	0	5	10	20	100	p^1^
Healthy (n=30)	5.97±0.12	6.34±0.63	7.19±0.78	7.68±0.73	8.83±0.79	0.001
SLE (n=30)	7.69±0.08	8.89±0.81	10.05±0.97	12.27±0.76	13.98±0.74	0.001
RA (n=30)	8.19±0.27	7.29±0.64	6.89±0.73	8.90±0.60	10.01±0.63	0.001
p^2^	0.001	0.001	0.001	0.001	0.001	
p^3^	0.001	0.001	0.352	0.001	0.001	
p^4^	0.001	0.001	0.001	0.001	0.001	

**Figure 2:**
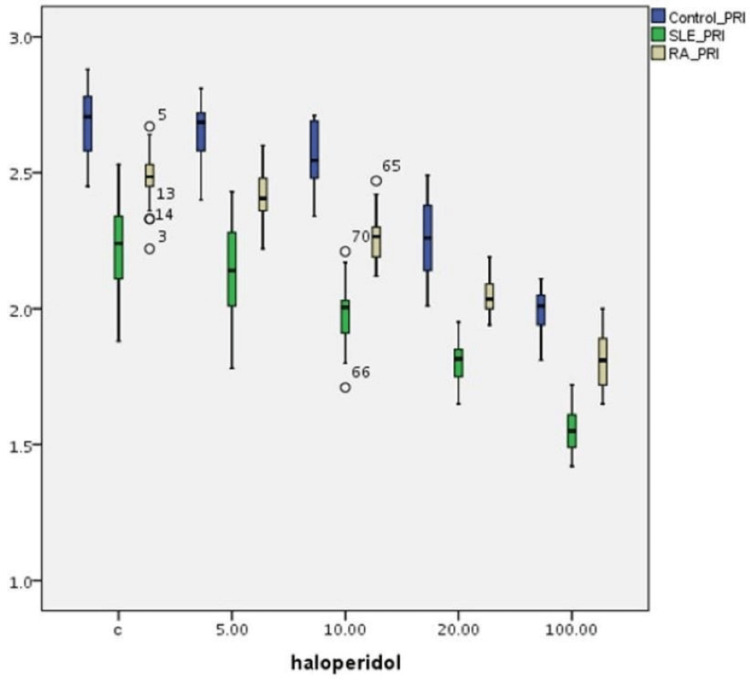
Haloperidol’s (μg/mL) effect on PRI index (PRI±SE) variations in human T-lymphocyte cultures from healthy individuals, SLE, and RA patients PRI: proliferation rate index; SLE: systemic lupus erythematosus; RA: rheumatoid arthritis

Table 2 summarizes the effect of haloperidol concentrations on PRI index variations on T-cultured lymphocytes of all three groups (Figure 2). There is a statistically significant difference in PRI frequencies between (a) the control and the SLE group (p=0.001), (b) the control and the RA group (p=0.001), and (c) the SLE and RA group (p=0.001) without haloperidol. PRI differences between these groups are also statistically significant (p=0.001) for various haloperidol concentrations. A statistically significant dose-dependent reduction in PRI frequency is observed in all three groups (p=0,001).

**Table 2:** Effect of haloperidol on PRI frequency (PRI±SE) in T lymphocyte cultures of SLE, RA patients, and healthy donors p^1^ represents the p-value for the Kruskal-Wallis test, which was performed to evaluate the dosage effect of haloperidol on the PRI index for all groups, p^2^ represents the p-value for the Mann-Whitney U test for the comparison of the PRI index between control and SLE group for various haloperidol concentrations, p^3^ represents the p-value for the Mann-Whitney U test for the comparison of the PRI index between control and RA group for various haloperidol concentrations, p^4^ represents the p-value for the Mann-Whitney U test for the comparison of the PRI index between RA and SLE group for various haloperidol concentrations. PRI: proliferation rate index; SCE: sister chromatid exchange; SE: standard error; SLE: systemic lupus erythematosus; RA: rheumatoid arthritis

Haloperidol (μg/mL)	0	5	10	20	100	p^1^
Healthy (n=30)	2.69±0.020	2.65±0.11	2.56±0.12	2.25±0.13	1.99±0.07	0.001
SLE (n=30)	2.23±0.028	2.15±0.16	1.97±0.11	1.81±0.08	1.55±0.08	0.001
RA (n=30)	2.48±0.016	2.41±0.09	2.25±0.09	2.04±0.06	1.81±0.09	0.001
p^2^	0.001	<0.001	0.001	<0.001	<0.001	
p^3^	0.001	<0.001	0.001	<0.001	<0.001	
p^4^	0.001	<0.001	0.001	0.001	<0.001	

**Figure 3:**
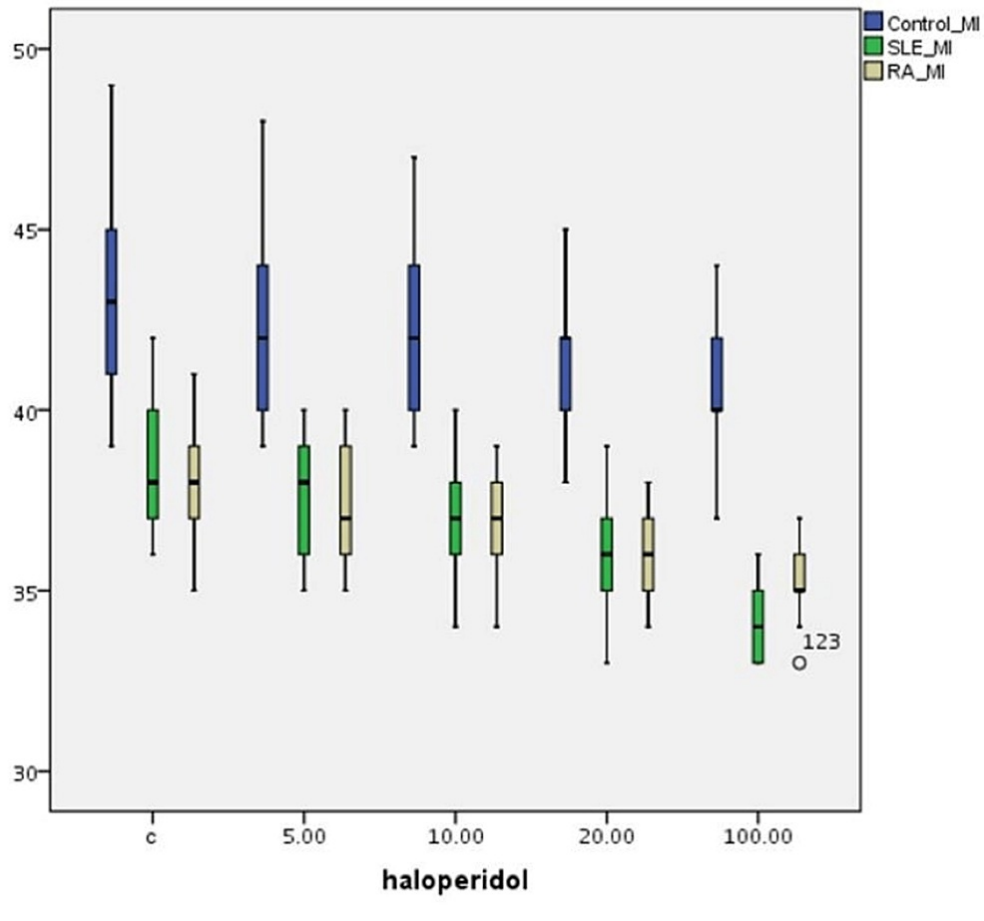
Haloperidol’s (μg/mL) effect on MI index (MI±SE) variations in human T-lymphocyte cultures from healthy individuals, SLE, and RA patients MI: mitotic index; SLE: systemic lupus erythematosus; RA: rheumatoid arthritis

Table 3 summarizes the effect of the haloperidol concentrations on MI index variations on T-cultured lymphocytes of all three groups (Figure 3). A statistically significant difference is observed in MI frequencies between (a) the control and the SLE group (p=0.001) and (b) the control and the RA group (p=0.001) without haloperidol, whereas the difference between the SLE and the RA group is not statistically significant (p=0.086). MI differences between the control group and patient groups are statistically significant (p=0.001) after the effect of various haloperidol concentrations. However, MI differences between SLE and RA groups are not statistically significant for haloperidol concentrations 5, 10, and 20μg/mL. For the highest haloperidol concentration, MI values are different at a statistically significant level (p=0.001). Moreover, a statistically significant dose-dependent reduction in MI frequency is observed in all three groups (p=0.001).

**Table 3:** Effect of haloperidol on MI frequency (MI±SE) in T lymphocyte cultures of SLE, RA patients, and healthy donors p^1^ represents the p-value for the Kruskal-Wallis test, which was performed to evaluate the dosage effect of haloperidol on the MI index for all groups, p^2^ represents the p-value for the Mann-Whitney U test for the comparison of the MI index between control and SLE group for various haloperidol concentrations, p^3^ represents the p-value for the Mann-Whitney U test for the comparison of the MI index between control and RA group for various haloperidol concentrations, p^4^ represents the p-value for the Mann-Whitney U test for the comparison of the MI index between RA and SLE group for various haloperidol concentrations. MI: mitotic index; SE: standard error; SLE: systemic lupus erythematosus; RA: rheumatoid arthritis

Haloperidol (μg/mL)	0	5	10	20	100	p^1^
Healthy (n=30)	42.90±0.44	42.53±2.25	42.30±2.22	41.37±1.67	39.49±2.79	0.001
SLE (n=30)	38.53±0.31	37.83±1.70	36.90±1.66	35.90±1.34	34.03±0.96	0.001
RA (n=30)	37.73±0.27	37.37±1.47	36.70±1.49	35.87±1.10	35.23±0.93	0.001
p^2^	0.001	0.001	0.001	0.001	0.001	
p^3^	0.001	0.001	0.001	0.001	0.001	
p^4^	0.001	0.235	0.722	0.814	0.001	

Table 4 illustrates the values and statistical significance of correlation rates between SCEs, PRI, and MI. For the control group, a statistically significant negative correlation is observed between SCEs and PRI (r= -0.684, p<0.001) and between SCEs and MI (r= -0.275, p=0.001). A statistically significant positive correlation is also observed between PRI and MI (r=0.245, p=0.002). For the SLE group, a statistically significant negative correlation is observed between SCEs and PRI (r= -0.827, p<0.001) and between SCEs and MI (r= -0.686, p<0.001). A statistically significant positive correlation is also observed between PRI and MI (r=0.733, p<0.001). For the RA group, a statistically significant negative correlation is observed between SCEs and PRI (r= -0.587, p<0.001) and between SCEs and MI (r= -0.367, p<0.001). A statistically significant positive correlation is also observed between PRI and MI (r= 0.579, p<0.001).

**Table 4:** Values and statistical significance of correlation rates between SCE, PRI, and MI ^1^Spearman's rho SCE: sister chromatid exchange; PRI: proliferation rate index; MI: mitotic index; SLE: systemic lupus erythematosus; RA: rheumatoid arthritis

Correlation	Value	p^1^
SCEs-PRI (Healthy)	-0.684	<0.001
SCEs-MI (Healthy)	-0.275	0.001
PRI-ΜΙ (Healthy)	0.245	0.002
SCEs-PRI (SLE)	-0.827	<0.001
SCEs-ΜΙ (SLE)	-0.686	<0.001
PRI-ΜΙ (SLE)	0.733	<0.001
SCEs-PRI (RA)	-0.587	<0.001
SCEs-ΜΙ (RA)	-0.367	<0.001
PRI-ΜΙ (RA)	0.579	<0.001

## Discussion

In the present study, an interesting cytogenetic behavior of haloperidol is observed. Haloperidol in vitro seems to be both cytotoxic and cytostatic in a dose-dependent way for T lymphocytes of healthy individuals and SLE patients. Moreover, at low concentrations (5 and 10μg/mL), haloperidol seems to have a cytoprotective effect with SCE reduction for T lymphocytes of RA patients. For higher concentrations tested, haloperidol seems to have cytotoxic behavior in the same group. It is also cytostatic in a dose-dependent manner for RA patients’ lymphocytes. In addition, T lymphocytes from SLE patients seem to be affected at lower haloperidol doses compared to T lymphocytes of healthy donors. Differences in the three cytogenetic indices are also observed in patient groups before the effect of haloperidol compared to those of the control group. This is probably related to DNA damage implicated in the pathogenesis of RA and SLE and to the lymphocytes’ exposure to patients’ standard treatment.

Haloperidol’s cytotoxic effects on T lymphocytes from healthy individuals are probably through inducing oxidative stress via reactive oxygen species (ROS) production and depletion of T lymphocytes' antioxidant enzymes [1]. Dopamine is a potent activator of effector T lymphocytes as they express functional dopamine receptors (D1R-D5R). Still, dopamine acts differently in different T lymphocyte subsets, in activated T lymphocytes, and in T lymphocytes from patients with autoimmune-neurodegenerative diseases [13]. Chronic treatment with haloperidol or treatment with hypertheraputic doses of the drug (over 20μg/mL) correlates to excessive ROS production [1,13] because of dopamine’s turnover and a decline in glutathione’s amount via modulation of proteins involved in the synthesis of selenocysteine in a dose depended on the way [1,14]. Moreover, haloperidol may induce DNA methylation and caspase-3 activity and MI reduction resulting in excessive DNA fragmentation, apoptosis, and dose-dependent alterations in DNA synthesis, contributing to its cytostatic behavior [1].

Although previews in vitro studies did not show SCEs elevation in lymphocytes from patients with RA compared to healthy individuals [15,16], DNA damage seems to be a cornerstone in RA pathogenesis [17,18]. Soultiotis et al. showed that DNA damage and inflammation are connected in RA, but the mechanism of DNA damage and genomic instability is not fully understood [18]. Oxidative stress and ROS production are the leading etiology for increased DNA damage in RA and many autoimmune diseases like SLE [18]. Reduced DNA repair efficiency and alterations in chromatin structure contribute to RA’s endogenous DNA damage [18]. In the present study, SCE elevation in the RA group, compared to both the control and SLE groups probably, represents methotrexate’s effect on T lymphocytes [19] and the increased median age of the RA group compared to both the control and SEL groups. Most RA patients are treated with methotrexate in the present study (24/30). Increased age correlates with SCE induction [6,11,12]. High doses of haloperidol (20, 100μg/mL) induce DNA damage (SCEs induction) and cytostatic behavior (PRI and MI elevation) via oxidative stress induction as in healthy individuals [1]. On the other hand, haloperidol in lower doses (5, 10μg/mL) may act differently not only in D2 receptors of RA patients’ lymphocytes [1,8,13] but also in a D2 independent manner that affects a novel form of cell-regulated death characterized by iron-dependent lipid peroxidation called ferroptosis [20,21]. T lymphocytes’ dopamine receptors differ between cells from RA patients compared to healthy individuals [13]. D2R antagonism with haloperidol in small doses may act in an antioxidant way and do not exert cytotoxicity [1,13]. In addition, haloperidol may directly influence DNA repair proteins, actin cytoskeleton, and Rho-GTPase signaling affecting DNA repair indirectly [14] via modulating cell cycle arrest and Ras-related C3 botulinum toxin substrate 1 (Rac1)/RhoA interaction [22]. MI stabilization of the RA group compared to the SLE group for lower haloperidol concentrations indicates such a mechanism of action with unaffected DNA synthesis. Furthermore, Hirata et al. showed that low doses of haloperidol can inhibit ferroptosis by reducing the accumulation of lysosomal ferrous ions [20]. This leads to reduced production of intracellular ROS, inhibition of cell death [21], and alterations in DNA damage response and repair (DDR/R) mechanism [22]. DDR/R elements such as p53 (which is underexpressed in RA lymphocytes) [21] play a crucial role in ferroptosis regulation [23]. Ferroptosis inhibitions by low dose haloperidol could upregulate p53 expression and promote SCE reduction as p53 binds to the replication fork and ensures a proper replication start [24].

SLE patients have difficulty maintaining genome stability and DNA repair mechanisms [25]. Previous cytogenetic studies in SLE patients confirm our observation of cultured T lymphocytes from SLE patients having higher SCE frequencies compared to healthy individuals [15]. T lymphocytes from the SLE group are more sensitive to oxidative stress compared to the control group and RA group due to superoxide dismutase decreased activity in SLE and the defection of nucleotide excision repair and DNA double-strand breaks repair mechanisms that lead to apoptosis [25]. In addition, a lot of DDR/R proteins are targets of autoantibodies in SLE and are related to DNA double-strand breaks repair and SCE induction [26]. A strong correlation between DNA damage and increased apoptosis rate is observed in SLE [26] as observed in the present study (Table 4). SCE elevation compared to both the control group and the RA group is probably due to increased ROS production and lack of glutathione even in lower haloperidol dosage [1], which leads to increased cytostaticity combined with corticosteroid use. Furthermore, ferroptosis in SLE happens only in neutrophils but not in monocytes or lymphocytes, a phenomenon likely due to the relatively lower levels of glutathione peroxidase 4 (Gpx4) in neutrophils [27].

Limitations of the study

The limitations of the study have to be taken into consideration to understand its results. Sample heterogeneity concerning population age and sex, lack of correlation with clinical indices and treatment, acute dosing of haloperidol, and in vitro study conduction are the most important limitations. Furthermore, molecular cytogenetic assays were not conducted, markers of oxidative stress were not measured, only a single lymphocyte population was tested, T lymphocyte subpopulations were not defined, and cytokine profile was not analyzed. Finally, our study lacks in vivo data for haloperidol’s cytogenetic behavior in patients with SLE and RA who are treated with haloperidol.

## Conclusions

In conclusion, haloperidol seems cytotoxic and cytostatic in a dose-dependent manner for lymphocytes of SLE patients and control groups but lymphocytes from the RA patient group are affected differently. No cytotoxic properties of haloperidol are noticed for the lowest concentrations (5,10μg/mL). On the contrary, haloperidol may have a protective role for lymphocyte DNA in RA patients at these concentrations. For higher concentrations, a cytostatic behavior is noticed. Haloperidol has cytostatic dose-dependent effects on cultured lymphocytes from RA patients too.

Oxidative stress, DNA damage control system, and probably ferroptosis play an important role, and this cytogenetic behavior needs further in vitro and in vivo investigation in other cell lines in combination with molecular cytogenetic techniques and in vivo in SLE and RA patients that are treated with haloperidol.
